# Accuracy of invasive arterial pressure monitoring in cardiovascular patients: an observational study

**DOI:** 10.1186/s13054-014-0644-4

**Published:** 2014-11-30

**Authors:** Stefano Romagnoli, Zaccaria Ricci, Diego Quattrone, Lorenzo Tofani, Omar Tujjar, Gianluca Villa, Salvatore M Romano, A Raffaele De Gaudio

**Affiliations:** Department of Anesthesia and Intensive Care, University of Florence, Azienda Ospedaliero-Universitaria Careggi, Florence, Italy; Department of Pediatric Cardiac Surgery, Bambino Gesù Children’s Hospital, Rome, Italy; Department of Neurosciences, Psychology, Drug Research and Child Health, University of Florence, Florence, Italy; Department of Heart and Vessels, University of Florence, Azienda Ospedaliero-Universitaria Careggi, Florence, Italy

## Abstract

**Introduction:**

Critically ill patients and patients undergoing high-risk and major surgery, are instrumented with intra-arterial catheters and invasive blood pressure is considered the “gold standard” for arterial pressure monitoring. Nonetheless, artifacts due to inappropriate dynamic response of the fluid-filled monitoring systems may lead to clinically relevant differences between actual and displayed pressure values. We sought to analyze the incidence and causes of resonance/underdamping phenomena in patients undergoing major vascular and cardiac surgery.

**Methods:**

Arterial pressures were measured invasively and, according to the fast-flush Gardner’s test, each patient was attributed to one of two groups depending on the presence (R-group) or absence (NR-group) of resonance/underdamping. Invasive pressure values were then compared with the non-invasive ones.

**Results:**

A total of 11,610 pulses and 1,200 non-invasive blood pressure measurements were analyzed in 300 patients. Ninety-two out of 300 (30.7%) underdamping/resonance arterial signals were found. In these cases (R-group) systolic invasive blood pressure (IBP) average overestimation of non-invasive blood pressure (NIBP) was 28.5 (15.9) mmHg (*P* <0.0001) while in the NR-group the overestimation was 4.1(5.3) mmHg (*P* <0.0001). The mean IBP-NIBP difference in diastolic pressure in the R-group was −2.2 (10.6) mmHg and, in the NR-group −1.1 (5.8) mmHg. The mean arterial pressure difference was 7.4 (11.2) mmHg in the R-group and 2.3 (6.4) mmHg in the NR-group. A multivariate logistic regression identified five parameters independently associated with underdamping/resonance: polydistrectual arteriopathy (*P* =0.0023; OR = 2.82), history of arterial hypertension (*P* =0.0214; OR = 2.09), chronic obstructive pulmonary disease (*P* =0.198; OR = 2.61), arterial catheter diameter (20 vs. 18 gauge) (*P* <0.0001; OR = 0.35) and sedation (*P* =0.0131; OR = 0.5). The ROC curve for the maximal pressure–time ratio, showed an optimum selected cut-off point of 1.67 mmHg/msec with a specificity of 97% (95% CI: 95.13 to 99.47%) and a sensitivity of 77% (95% CI: 67.25 to 85.28%) and an area under the ROC curve by extended trapezoidal rule of 0.88.

**Conclusion:**

Physicians should be aware of the possibility that IBP can be inaccurate in a consistent number of patients due to underdamping/resonance phenomena. NIBP measurement may help to confirm/exclude the presence of this artifact avoiding inappropriate treatments.

## Introduction

Arterial pressure measurement represents a mandatory step in the evaluation of patients’ hemodynamics because it gives primary information about the performance of the cardiovascular system and tissue perfusion [1]. In every clinical condition, arterial pressure monitoring should hence be as accurate as possible [2,3]. For this reason, in critically ill patients and in patients undergoing high-risk and major surgery, direct intra-arterial pressure measurement (invasive blood pressure, IBP) is considered the gold standard, allowing beat-by-beat measures even in patients receiving inotropic or vasoactive drugs, or in cases of abrupt changes in blood volume or arterial tone, or those with arrhythmias [3-5]. The alternative to IBP monitoring is the non-invasive (NIBP) system (oscillometric technique). However, NIBP measurement is not continuous and, during hemodynamic instability, severe hypotension, in conditions of increased arterial stiffness and in obese patients, this technique is expected to be less accurate than the invasive one [2,4-8]. These considerations may be disregarded in case of IBP system calibration errors, altered pulse travelling (arterial dissection or stenosis) and artifacts due to movement or inappropriate dynamic response of the fluid-filled monitoring systems (overdamping and underdamping) [9,10]. In 2011, we demonstrated that in patients undergoing vascular surgery, the incidence of arterial pressure artifacts due to underdamping of the pressure transducer (or resonance artifact) was as high as 34.9% [10]. The aim of this study was to identify the global incidence of IBP measurement errors due to underdamping/resonance artifacts in three different categories of patients: patients undergoing vascular surgery, patients undergoing cardiac surgery, and patients admitted to a cardiac surgery ICU. Additional aims included error quantification through the comparison of IBP and NIBP measurements, identification of clinical and pharmacological independent predisposing factors or other variables associated with underdamping/resonance and analysis of the threshold value of the maximal pressure-time ratio (pressure rise during systole) (dP/dt_MAX_) possibly suggesting underdamping/resonance of the arterial pressure signal.

## Materials and methods

A prospective observational cohort study was conducted. The study was approved by the hospital ethics committee of the Azienda Ospedaliero-Universitaria Careggi - University of Florence, and informed consent was obtained by all participants. All consecutive patients with indwelling radial arterial catheter as standard practice in vascular surgery and cardiac surgery operating theatres and cardiac surgery ICU were enrolled in the study. The exclusion criteria were: age <18 years, body mass index ≥30 Kg/m^2^, any type of arrhythmia or hemodynamic instability (>5 mmHg variation in mean blood pressure) during the period of data collection, presence of tissue edema, an artery line different to the left or right radial artery, overdamped arterial signals (according to a fast-flush test), and the inability to measure NIBP in the same arm as that in which the arterial line was placed. Data acquisition has been performed by the investigators prior to anesthesia induction, during surgery, in the ICU, with or without sedation. None of the investigators was involved in the clinical management of the patients.

**Figure 1:**
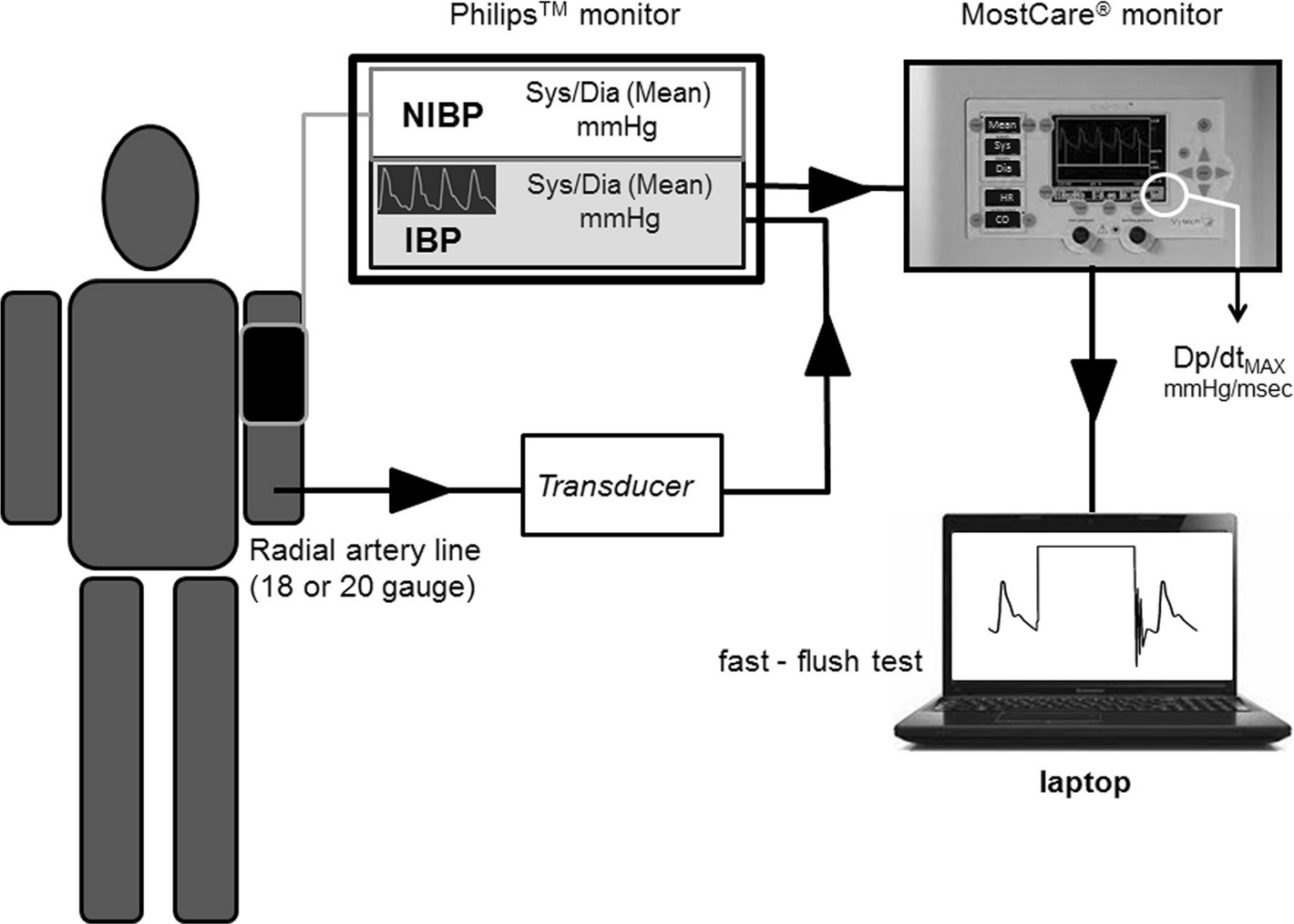
**Schematic representation of the patient’s connection to the Philips™ monitor for invasive blood pressure (IBP) and non-invasive blood pressure (NIBP) monitoring.** MostCare® is connected via an analog output for the continuous recording, at 1,000 Hz, of the systemic arterial pressure waves and DP/dt_MAX_ computation. The analog pressure signals are recorded on a personal computer for fast-flush test registration and analysis. Sys, systolic; Dia, diastolic; DP/dt_MAX_: maximal pressure-time ratio.

**Figure 2:**
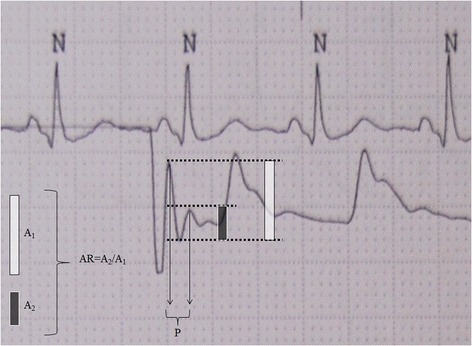
**Fast-flush test.** Amplitude ratio (AR): A_2_ (3 mm)/A_1_ (7 mm) = 0.43. The corresponding damping coefficient is 0.28 [9]. P identifies the period (peak-to-peak distance) necessary for the natural frequency calculation: paper speed/P. In the example, 25 (mm/sec)/2 mm = 12.5 Hz; these data can be then plotted into the diagram showed in Figure 3. N: normal QRS complex.

**Figure 3:**
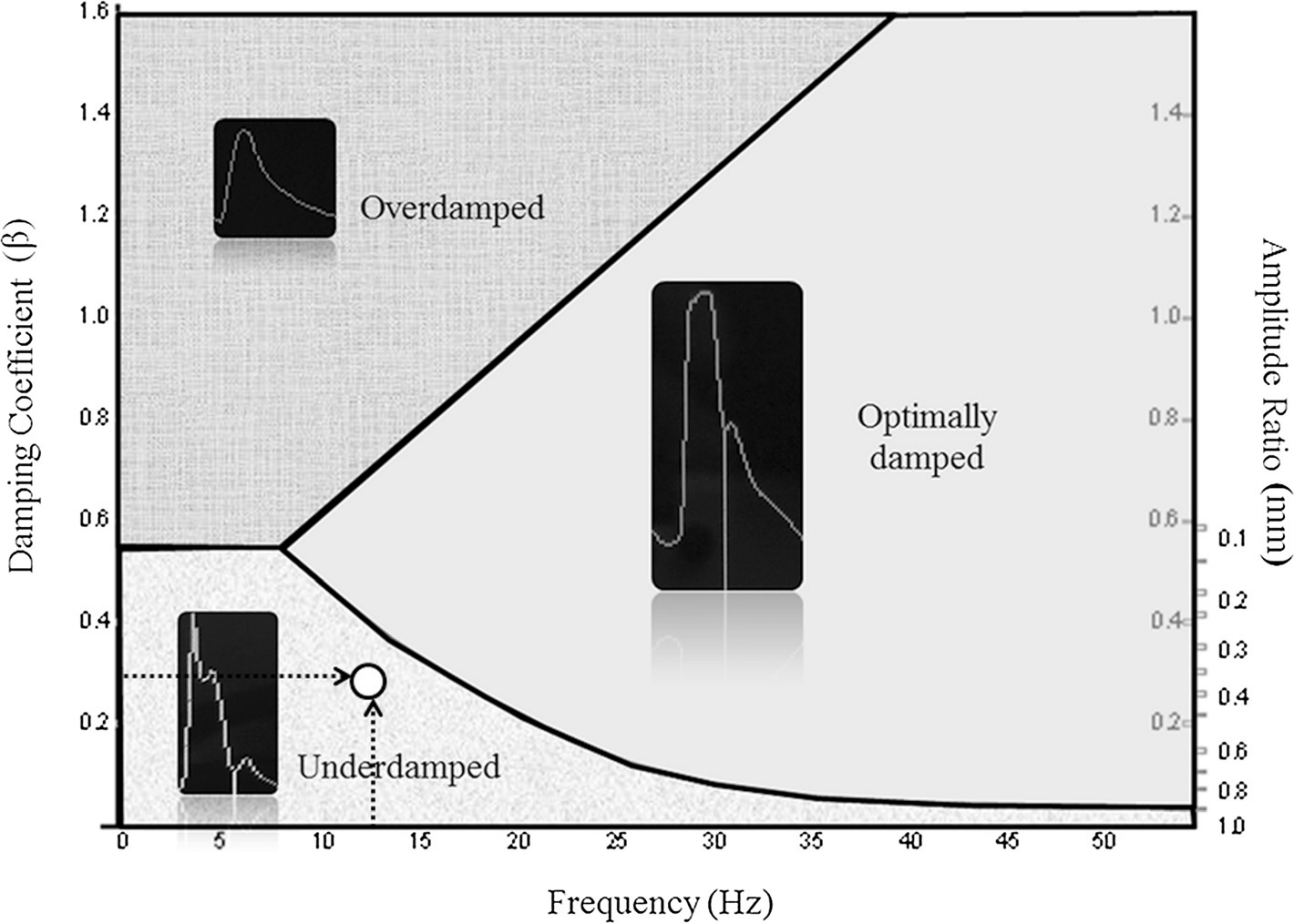
**Diagram showing the three areas for underdamped, overdamped, and optimally damped blood pressure signal.** The black arrows indicate the natural frequency and the damping coefficient of the example showed in Figure 2. The white point identifies an underdamped/resonant arterial pressure signal.

A standard procedure was used for all measurement collection. First, the arterial pressure transducer was leveled and zeroed to the intersection of the anterior axillary line and the fifth intercostal space. The investigators then purged the system of any air bubble with a dedicated inflated flush system set at 300 mmHg. Second, invasive systolic blood pressure (Sys-IBP), diastolic blood pressure (Dia-IBP), and mean blood pressure (Mean-IBP) were measured by means of a radial artery catheter (Leadercath Arterial polyethylene catheter - 18 gauge, 10 cm length, 0.8 mm internal diameter × 1.2 mm external diameter or 20 gauge, 8 cm length, 0.6 mm internal diameter × 0.9 mm external diameter; Vygon, Ecouen, France), connected to a disposable pressure transducer (Package transducer Edwards; VAMP Plus system; Edwards Lifesciences, Irvine, CA, USA) and measured with a Philips MP60 IntelliVue monitor (Philips Medical System; Best,The Netherlands). The signal was then directed via the analogic output to a MostCare® pulse contour hemodynamic monitoring system (Vygon, Vytech Health, Padova, Italy) in order to measure the dP/dt_MAX_. This ratio represents the maximal rate of pressure change over time between two consecutive points during the systolic upstroke recorded at 1000 Hz: in a previous study [10] this parameter was shown to be significantly higher in underdamped/resonant signals that in non-affected waveforms. Moreover, the analog pressure signals were digitized using a multifunction board (Multifunction Analog and Digital Board RTI-800, Analog-Devices, Norwood, MA, USA), and recorded on a personal computer (LTE5000, Compaq, Houston, TX, USA) for fast-flush test registration and analysis [11] (Figure 1). The fast-flush test is the only one that allows clinicians to evaluate, the appropriateness of the dynamic response of the blood pressure measuring system at the bedside. The Fn and the damping coefficient (β) must be measured. The test is described elsewhere in details [9]: briefly, it is performed by flushing saline with high pressure (300 mmHg) via the flush system of the transducer. This, generates an undershoot and overshoot of waves that will decay exponentially in accordance with the β. The natural frequency (Fn) can be measured by dividing the paper speed (for example, 25 mm/sec) by the wavelength or period (peak to peak distance in mm) generated by the flush (Figure 2). Damping (anything that reduces energy in an oscillating system) will reduce the amplitude of the oscillations and some degree of damping is required in all systems (for example, friction in the fluid pathway). The β can be derived from the amplitude ratio (AR) of two consecutive resonant waves. AR is calculated by dividing the smaller wave (second) by the higher one (first) (Figure 2). Once the AR is measured, the corresponding β is then taken from charts [9]. Finally, the Fn and the AR or the corresponding β can be plotted in a specific graph that shows three areas: adequate dynamic response, overdamping, underdamping [9] (Figure 3).

Blood pressure values were recorded for thirty seconds each and the average value taken for analysis. Finally, the systolic (Sys)-NIBP, diastolic (Dia)-NIBP, Mean-NIBP values were measured and recorded (an average of four) [12] immediately following each invasive data collection, using the same arm as that in which the indwelling catheter was placed. The appropriate relationship between cuff size and upper arm circumference was respected according to current guidelines [6]. Mean blood pressure was delivered by each monitor according to the manufacturer’s algorithm, avoiding the application of any formula based on systolic and diastolic values.

Each patient was then assigned to one of two groups depending on the presence (R-group) or absence (NR-group) of resonance/underdamping identified by the fast-flush test [9]. In addition, a series of clinically relevant variables were registered in order to evaluate a possible correlation with underdamping/resonance artifact.

### Statistical analysis

All data were analyzed using Stats Direct (Ver.2.5.8, Bonville Chase Altrincham Cheshire, UK) and GraphPad (Ver. Prism 4.0; San Diego, CA, USA). The single-sample Kolmogorov-Smirnov test was used to test all data for normality. Continuous variables were expressed as mean (SD) or median with interquartile range where applicable. For dichotomous data, percentages were calculated. IBP and NIBP values were compared using the two-sided paired *t*-test in both the R-group and NR-group. Pairwise comparison was performed using the regression-based Bland-Altman technique, which models the mean and SD of the blood pressure differences as a function of the averaged measurements obtaining the bias (mean difference between methods) and limits of agreement (LoA) (calculated as the bias × 1.96 SD of the measurement differences) [13]. The unpaired *t*-test was used for the identification of differences in norepinephrine infusion between the R- and NR-groups. A two-sided *P*-value <0.05 was considered to be statistically significant.

In order to define the possible clinical/pharmacological or technical factors associated with the resonance phenomenon, we performed multivariable logistic regression analysis (backward selection) (covariates were retained in the model if the *P*-value was <0.1) with underdamping/resonance as the dependent variable. Co-linearity between variables was excluded before modeling and none of the covariates was co-linear. The Hosmer and Lemeshow goodness-of-fit test was performed and the odds ratio (OR) and 95% CI was computed.

Sample size was calculated taking into consideration that in a previous study from our department [10] systolic pressure of arterial signals affected by underdamping/resonance had an SD of 44.4 mmHg and the difference between IBP with and without underdamping/resonance was 15 mmHg. In order to repeat the same results, we calculated that at least 70 patients in the resonant group should have been enrolled to guarantee a 90% power detecting a mean difference between IBP and NIBP of 15.61 mmHg with a significance level (alpha) of 0.05 (one-tailed). Considering that in the reference paper, resonant patients were 30% of the overall population, in order to enroll all the required patients with underdamped/resonant arterial signals we calculated that at least 235 patients had to be enrolled.

## Results

During the study period, 348 patients were consecutively evaluated; of these, 48 were excluded for the following reasons: age <18 years (2 patients), body mass index ≥30 Kg/m2 (10 patients), any type of arrhythmia or hemodynamic instability (>5 mmHg variation in mean blood pressure; 32 patients), or artery line other than left or right radial (4 patients). No patient was excluded for overdamping. Three hundred patients were definitively enrolled in the study. They were divided as follows: 1) patients undergoing vascular surgery for arterial disease (percutaneous angioplasty and stenting for peripheral artery disease, carotid artery angioplasty and stenting, open surgery carotid endarterectomy, abdominal endovascular and open aortic repair) (n = 70; 23.3%); 2) patients undergoing cardiac surgery (off-pump and on-pump coronary artery bypass graft surgery, valvular surgery, aortic surgery, combined interventions) (n = 97; 32.3%); 3) patients admitted to cardiac surgery ICU (different from the previous group of patients) (n = 133; 44.4%) (Table 1). Patients’ characteristics are summarized in Table 2.

**Table 1:** **Incidence of underdamping/resonance**

	**Overall,** **number (%)**	**R-group,** **number (%)**	**NR-group,** **number (%)**
Overall	300 (100)	92 (30.7)	208 (69.3)
Vascular surgery theater	70 (23.3)	20 (21.7)	50 (24)
Cardiac surgery theater	97 (32.3)	27 (29.4)	70 (33.6)
Cardiac surgery ICU	133 (44.4)	45 (48.9)	88 (42.4)

R-group, patients with arterial signals affected by underdamping/resonance; NR-group, patients without arterial pressure artifact.

**Table 2:** **Patients’ characteristics**

	**Age, yr (SD); range**	**Gender, male, number/total; %**	**BSA,m** ^**2**^ **(SD); range**	**LV**_**EF**_ **-, % (SD); range**	**HR, beats/minute (SD); range**
Overall	69 (11); 21 to 87	201/300; 67	1.84 (0.18); 1.3 to 2.55	55 (11); 20 to 76	77.4 (13.9); 37 to 140
Vascular surgery theater	72 (8); 51 to 87	56/70; 81.1	1.82 (0.15); 1.3 to 2.16	60 (7) 30 to 75	67.1 (13.3); 37 to 98
Cardiac surgery theater	67 (13); 21 to 87	67/97; 69	1.86 (0.19); 1.44 to 2.51	54 (11); 20 to 75	78.2 (13.4); 43 to 115
Cardiac ICU	68 (11); 25 to 86	79/133; 59	1.84 (0.19); 1.41 to 2.55	53 (11); 20 to 76	82 (11.7); 54 to 140

BSA, body surface area; LV_EF_, left ventricle ejection fraction; HR, heart rate.

**Figure 4:**
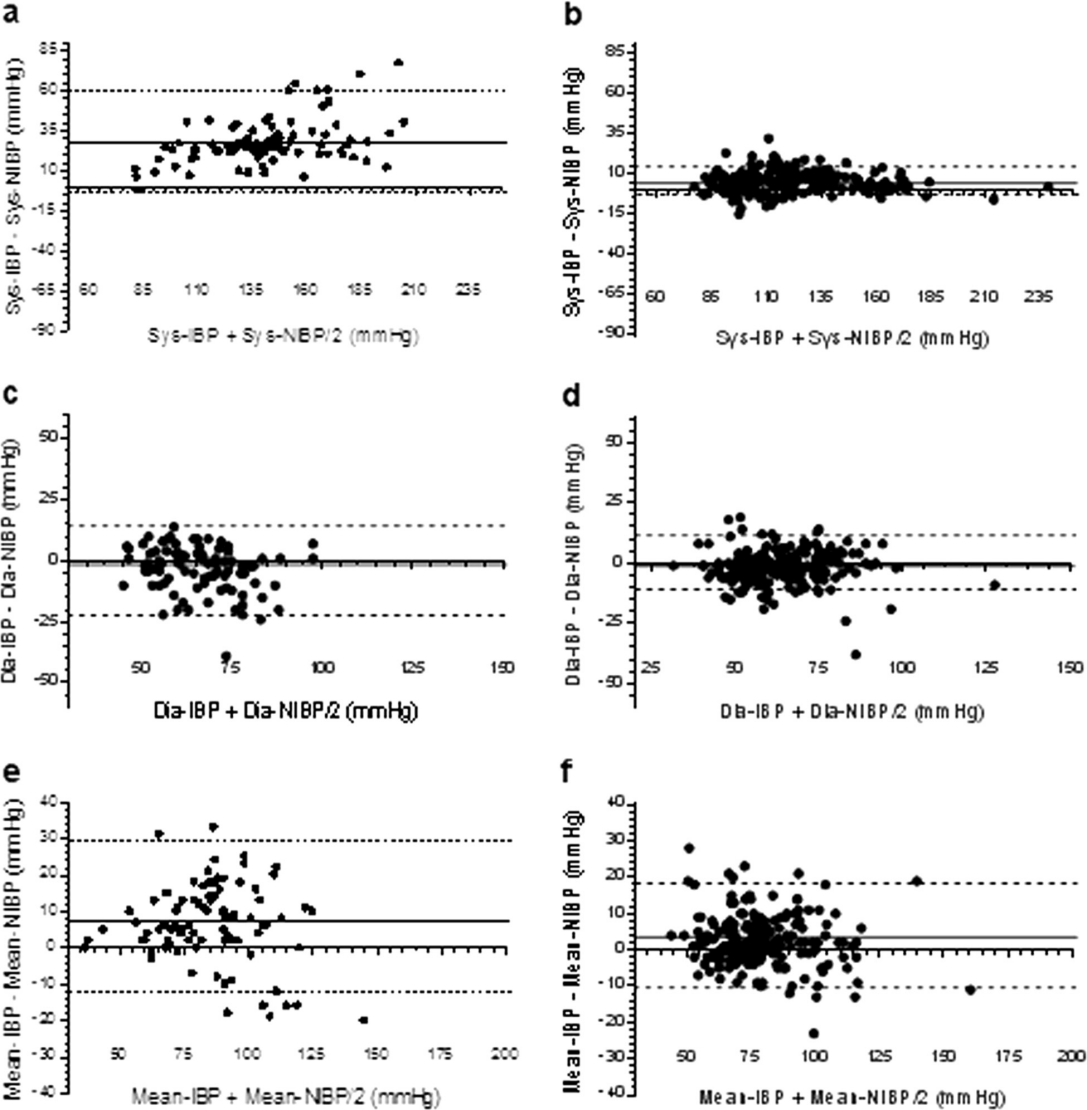
**Bland-Altman plots in the resonance (R-group) (a, c, e) and the non-resonance (NR)-group (b, d, f).** Sys, systolic; Dia, diastolic; IBP, invasive blood pressure monitoring; NIBP, non-invasive blood pressure monitoring.

A total of 11,610 IBP pulses and 1,200 NIBP measurements were performed, and the respective averages included in the analysis. The global incidence of underdamping/resonance was 30.7% (92 out of 300 patients) with a rather homogeneous distribution among the different settings (vascular surgery theatre: 30%; cardiac surgery theatre: 27.8%; cardiac surgery ICU: 33.8%). Fast-flush test for these patients resulted in an Fn of 15.9 (6.12) Hz with an AR of 0.39 (0.07) and β of 0.29 (0.05). In both the R-group and the NR-group, Sys-, Dia-, and Mean-BP measured by IBP were significantly different from NIBP values (*P* <0.05) (Table 3). The comparison of blood pressure by means of the Bland-Altman method (Tables 3 and 4; Figure 4) showed a mean Sys-IBP overestimation (bias) of 28.5 mmHg (range 2 to 77 mmHg; LoA −2.92 to 59.58 mmHg) in the R-group, while in the NR-group the overestimation was 4.1 mmHg (range −15 to 15 mmHg; LoA −6.19 to 14.40 mmHg). The bias in Dia-IBP was −2.2 (10.6) mmHg in the R-group (range −39 to −10 mmHg; LoA −23.42 to 14.81 mmHg) and −1.1 (5.8) mmHg in the NR-group (range −17 to 14 mmHg; LoA −12.5 to 10.3 mmHg), while it was 7.4 (11.2) mmHg (range −20 to 44 mmHg; LoA −14.98 to 29.50 mmHg) for Mean-IBP in the R-group and 2.3 (6.4) mmHg (range −13 to −20 mmHg; LoA −13.09 to 17.69 mmHg) in the NR-group.

**Table 3:** **Blood pressure measurements**

	**Overall**	**R-group**	**NR-group**
**Systolic IBP mmHg (SD); range**	134 (32); 78 to 241	157 (33); 82 to 241	124 (27); 78 to 240
**Systolic NIBP mmHg (SD); range**	122 (26); 76 to 238	129 (26); 76 to 191^a^	120 (27); 76 to 238^b^
**Δ Systolic BP mmHg (SD); range**	13 (14); −11 to 77	28 (16); 2 to 77	4 (5); −15 to 15
**Diastolic IBP mmHg (SD); range**	64 (13); 31 to 123	64 (12); 40 to 101	63 (13); 31 to 123
**Diastolic NIBP mmHg (SD); range**	66 (14); 32 to 132	69 (14); 43 to 98^C^	64 (13); 32 to 132^d^
**Δ Diastolic BP mmHg (SD); range**	2 (9); −27 to 82	−2 (11); −39 to 10	−1 (6); −17 to 14
**Mean IBP mmHg (SD); range**	83 (18); 37 to 155	90 (19); 37 to 135	81 (17); 46 to 155
**Mean NIBP mmHg (SD); range**	79 (19); 37 to 166	82 (21); 37 to 155^e^	78 (18); 41 to 166^f^
**Δ Mean BP mmHg (SD); range**	5 (9); −20 to 44	7 (11); −20 to 44	2 (6); −13 to 20

^a^Systolic IBP R-group versus Systolic NIBP R-group: *P* <0.0001; ^b^Systolic IBP NR-group versus Systolic NIBP NR-group: *P* <0.0001; ^C^Diastolic IBP R-group versus Diastolic NIBP R-group: *P* = 0.0002; ^d^Diastolic IBP NR-group versus Diastolic NIBP NR-group: *P* = 0.0018; ^e^Mean IBP R-group versus Mean NIBP R-group: *P* <0.0001; ^f^Mean IBP NR-group versus Mean NIBP NR-group: *P* <0.0001. Sys, systolic; IBP, invasive blood pressure monitoring; NIBP, non-invasive blood pressure monitoring; Dia, diastolic; Δ, difference; R-group, resonance group; NR-group, non-resonance group.

**Table 4:** **Comparison of blood pressure using the Bland-Altman method**

	**Bias, mmHg**	**Limits of agreement, mmHg**
R-group systolic IBP versus systolic NIBP	28.5	−2.92 to 59.58
NR-group systolic IBP versus systolic NIBP	4.1	−6.19 to 14.40
R-group diastolic IBP versus diastolic NIBP	−2.2	−23.42 to 14.81
NR-group diastolic IBP versus diastolic NIBP	−1.1	−12.5 to 10.3
R-group mean IBP versus mean NIBP	7.4	−14.98 to 29.50
NR-group mean IBP versus mean NIBP	2.3	−13.09 to 17.69

R-group, resonance group; NR-group, non-resonance group; IBP, invasive blood pressure monitoring; NIBP, non-invasive blood pressure monitoring.

**Figure 5:**
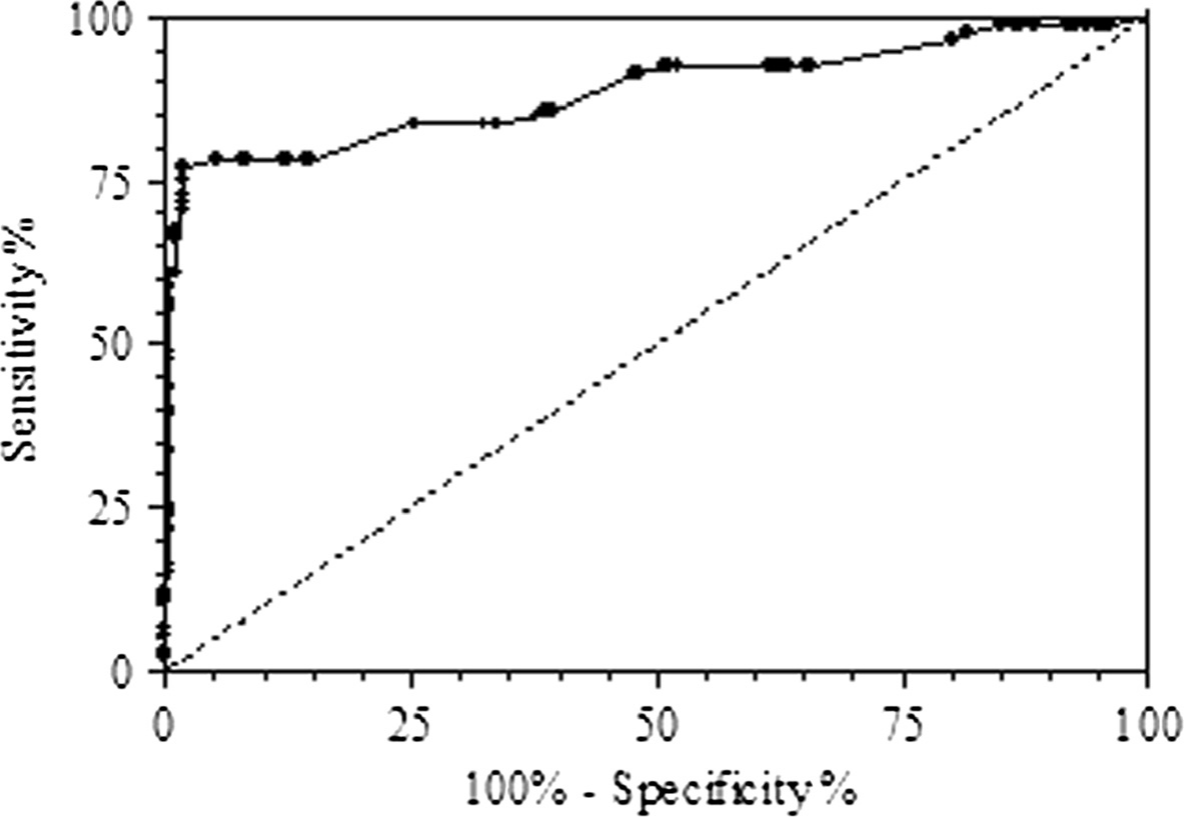
**Receiver operating characteristic curve for maximal pressure-time ratio (DP/dt**_**MAX**_ **)** **.**

Of 300 patients 71 (23.6%) received a continuous infusion of norepinephrine at a dose of 0.019 (0.043) μg/Kg/minute. The R-group received a lower norepinephrine dosage (0.01 (0.03) μg/Kg/minute;) than the NR-group (0.02 (0.04) μg/Kg/minute) (*P* = 0.066). No statistically significant differences were found in epinephrine dose (*P* = 0.15), arginine-vasopressin dose (*P* = 0.56), heart rate (*P* = 0.62) or ejection fraction of the left ventricle (preoperative measurement during routine risk stratification) (*P* = 0.19) when comparing the two groups. The multivariate logistic regression included a total of 18 parameters (3 anthropometric, 12 clinical, 2 pharmacologic, and 1 technical) (Table 5). The final analysis identified five parameters independently associated with underdamping/resonance (Table 6): polydistrectual arteriopathy (*P* = 0.0023), history of arterial hypertension (*P* = 0.0214), chronic obstructive pulmonary disease (*P* = 0.0198), arterial catheter diameter (20- versus 18-gauge) (*P* <0.0001), and sedation (*P* = 0.0131). Heart rate (HR) was not correlated with underdamping/resonance even after division into three terciles (tercile-I including HR ≤73 b/minute, *P* = 0.23; tercile-II including HR >73 and ≤84 b/minute, *P* = 0.89; tercile-III including HR > 84 b/minute, *P* = 0.92). According to the OR, three of these parameters resulted from a predisposition to underdamping/resonance: polydistrectual arteriopathy (OR = 2.82), chronic obstructive pulmonary disease (OR = 2.61), and arterial hypertension (under drug therapy) (OR = 2.09). Two of them were protective: arterial catheter diameter (20- versus 18-gauge) (OR = 0.35) and sedation (OR = 0.5). Finally, the receiver operating characteristic (ROC) curve for the maximal pressure-time ratio (dP/dt_MAX_), showed an optimum selected cutoff point of 1.67 mmHg/msec with a specificity of 97% (95% CI: 95.13 to 99.47%), a sensitivity of 77% (95% CI: 67.25 to 85.28%), and an area under the ROC curve by extended trapezoidal rule of 0.88 (Figure 5).

**Table 5:** **Logistic regression - backward selection (18 parameters)**

**Variable**	***P*** **-value**
**Anthropometric**	
Age	0.7077
Sex	0.2404
Body surface area	0.4456
**Clinical**	
Polydistrectual arteriopathy	0.0025
Coronary artery disease	0.2651
Valvular disease	0.8716
Abdominal aortic aneurysm	0.6594
Carotid artery disease	0.3624
History of arterial hypertension	0.0836
Non-insulin dependent diabetes mellitus	0.1804
Chronic renal failure	0.7213
Chronic obstructive pulmonary disease	0.0082
Left ventricle ejection fraction	0.4889
Heart rate (beats/minute)	0.5753
Core temperature	0.1233
Pharmacologic	
Sedation	0.0078
Norepinephrine infusion	0.1142
Technical	
Catheter diameter (18- or 20-gauage)	<0.0001

**Table 6:** **Logistic regression - backward selection**

**Parameter**	***P*** **-value**	**Odds ratio**	**95% CI**
Polydistrectual arteriopathy	0.0023	2.82	1.45 to 5.50
Chronic obstructive pulmonary disease	0.0198	2.61	1.16 to 5.87
Arterial hypertension	0.0214	2.09	1.12 to 3.93
Arterial catheter 20- versus 18-gauge	<0.0001	0.35	0.21 to 0.58
Sedation	0.0131	0.5	0.29 to 0.86

## Discussion

Technical details of the physical properties of fluid-filled catheter-transducer systems are described elsewhere. Some information on this may nevertheless be useful in order to better understand the link between the predisposing or protecting factors and the artifact [10,14]. Briefly, once an artery has been cannulated, the pulse generated by the left ventricle and transmitted via the arterial tree flows from an indwelling catheter through fluid-filled capillary tubing. The transduced pressure causes displacement of the transducer membrane, converting blood pressure - a time-varying physiologic event - into an electrical signal via a resistive bridge. The electrical signal is amplified and then digitally displayed. For a faithful representation of the arterial pressure waveform and pressure values, the Fn of oscillation of the transducer system (the frequency of unforced vibrations or the frequencies at which an object tends to vibrate when disturbed in the absence of damping forces) must exceed the frequencies of the impulses generated by the cardiovascular system (harmonics). When one of the frequencies generated by the cardiovascular system overlaps the Fn of the transducer, the underdamping/resonance phenomenon occurs. Therefore, the Fn and the β (how quickly the system comes to rest after being disturbed) constitute the dynamic response characteristics of the monitoring system. These in turn determine the system’s ability to reproduce arterial waveform without distortion. The underdamping/resonance phenomenon can hence be defined as the tendency of a system to oscillate with greater amplitude at some frequencies than at others.

The results of the present study confirm our previous clinical observation that the occurrence of underdamping/resonance is present in about 30% of critically ill patients. This artifact is probably frequently overlooked but may have clinical relevance and may lead to inappropriate therapeutic approaches if the artifact is not recognized and corrected [7]. It must be emphasized that a scarce body of literature is currently evaluating the accuracy of IBP with modern transducers and monitors. On the other hand, according to our data, patients with a resonant arterial waveform according to Gardner’s test [9] appeared to have significantly different NIBP measurements that, in these conditions, may be considered the most reliable one [14].

In fact, the IBP-NIBP difference in NR patients was clinically irrelevant, indicating that NIBP might be considered a reliable and accurate method even in the ICU, with the exception of certain specific conditions (for example, obese patients, extreme hypotension, arrhythmias) [15,16]. As further confirmation of this, a recently published survey of blood pressure monitoring showed NIBP to be commonly used by a large number of ICU physicians in the United States [4]. In our cohort of patients, we found a rather homogeneous distribution of underdamping/resonance among the three groups, with Sys values being the most affected when compared to NIBP. In fact, in patients with underdamping/resonance, Sys-IBP severely overestimated Sys-NIBP with a bias as high as 28.5 mmHg, and ranging from 2 to 77 mmHg (Figure 4). The extent of the bias was less pronounced for Dia-IBP versus Dia-NIBP (−2.2 mmHg) but still consistent for Mean-IBP versus Mean-NIBP (7.4 mmHg). This fact is of utmost importance for physicians because several consensus statements and guidelines have recommended the optimization of both systolic and mean arterial pressure in order to improve tissue perfusion in the ICU [3,5,17]. In these conditions, the inaccurate measurement of arterial blood pressure may cause inappropriate interventions such as a lack of fluids and/or vasoactive/inotropic support in true hypotension, or the treatment of false systolic hypertensions with a consequent reduction in tissue perfusion pressure. A difference >10 mmHg with actual arterial pressure is clinically relevant, and >20 mmHg is clinically unacceptable [7]. Interestingly, in the NR-group, the bias of the Sys, Dia, and Mean BP between IBP and NIBP was very limited (4.1, −1.1 and 2.3 mmHg respectively). This concurs with existing literature [12,18].

In our cohort of patients, the multivariate analysis identified five conditions independently correlated with underdamping/resonance: three clinical conditions (chronic arterial hypertension, polydistrectual arteriopathy and chronic obstructive pulmonary disease (COPD)), one pharmacological state (sedation), and a technical parameter (arterial catheter diameter and length). We found that hypertensive arteriopathy is a predisposing factor for underdamping/resonance: this result confirms the findings of our previous work in vascular surgery patients [10]. It is currently uncertain what this strong association depends on: however, as it may be a consequence of the high stiffness of the arterial system that may contribute to the generation of high frequency harmonics eventually overlapping the Fn of the catheter-tubing-manometer system. In support of this, we found that polydistrectual arteriopathy and arterial hypertension were independently associated with underdamping/resonance artifacts with an OR of 2.82 (*P* = 0.0023) and 2.09 (*P* = 0.021) respectively. COPD has been found to be correlated with underdamping/resonance (OR = 2.61; *P* = 0.0198). This association may be explained with the hypothesis that arterial hypertension and atherosclerosis, both associated with underdamping/resonance and frequently affecting COPD patients [19], may have been missed in the medical history. On the contrary, sedation has a protective role (OR = 0.5; *P* = 0.013), as the reduction in vascular tone caused by a direct effect on the vessels’ smooth muscles and attenuation of the sympathetic nervous system limits the development of high frequency harmonics [10,20]. Finally, the length and, mainly, the diameter of the arterial catheter play a key role in the development of underdamping/resonance artifacts. This phenomenon can be clearly explained by looking at the following equation for the damping coefficient (β) [21]:

$$ \upbeta = 4\upmu /{\mathrm{r}}^3{\left(\uprho \mathrm{L}/\uppi \mathrm{E}\right)}^{1/2} $$

Here, β is the damping coefficient (entity of friction forces), μ is the viscosity of the fluid, r is the internal radius of the catheter, ρ is the density of the fluid, L is the length of the catheter, and E is the elasticity of the system. By increasing the internal radius (r) of the catheter (that is, by replacing a 20-gauge with an 18-gauge catheter), the damping coefficient (β) decreases significantly as it varies inversely to the cube of the radius. Similarly, by increasing the length of the catheter (L), β also increases. Moreover, as the physical properties of the catheter-tubing-manometer system determine the Fn according to the following formula [14]:

$$ \mathrm{F}\mathrm{n}=1/2\uppi\ \surd k/m $$

(where *k* is the elasticity and *m* is the mass), by changing the length and the diameter of the arterial cannula, the Fn changes as well.

Our data show a strong relationship between catheter diameter and length and underdamping/resonance, with smaller catheters demonstrating a highly protective effect (20- versus 18-gauge; OR = 0.35; *P* <0.0001). When an arterial line is put in place, this fact should certainly be taken into consideration. Equally, eventual fibrin deposition may, with time, increase β. Lambermont *et al*. developed, in an experimental setting, a mathematical transfer function that, by adjusting the natural frequency and the damping coefficient of the fluid-filled catheter system, may reconstruct the pressure waveform free of artifacts [22]. At a practical level, however, it is very important for the clinician to be aware of the high incidence of underdamped/resonant arterial waveforms in critically ill adult patients. Non-invasive systolic pressure measurement may be helpful to suspect and identify this artifact at the bedside. Newer hemodynamic monitors measure the dP/dt_MAX_ as an indicator of myocardial contractility [23,24]. In 2011, we observed that dP/dt_MAX_ in arterial signal affected by underdamping/resonance was significantly higher than in artifact-free waveforms [10]. In the present study, we performed a ROC curve analysis in order to identify a predicting value for underdamping/resonance. The ROC curve showed that a threshold value of 1.67 could discriminate an underdamped/resonant signal from a non-underdamped/non-resonant signal with a specificity of 97% (95% CI: 95.13 to 99.47%), a sensitivity of 77% (95% CI: 67.25 to 85.28%), and an area under the ROC curve by extended trapezoidal rule of 0.88. Therefore, a dP/dt_MAX_ greater than 1.67 may be helpful in identifying suspected underdamped/resonant signals prior to the performance of a fast-flush test. Moreover, in light of our results, the comparison of Sys-IBP and Sys-NIBP may have a strong predictive value for the identification of resonance-affected waveforms.

Finally, we acknowledge several weaknesses. First, the oscillometric method for NIBP measurement is not standardized. Algorithms for systolic and diastolic pressure estimation may differ from manufacturer to manufacturer, and even from device to device [6]. We used a modern and quite diffuse system; however, different monitors could have given different data to some extent. Second, severe hypotension is commonly recognized as limiting the accuracy of NIBP, although recent data call this statement into question [12]. In our observational study, systolic pressures <70 mmHg did not appear in the final analysis. Third, IBP and NIBP were not registered pairwise at precisely the same time but one momentarily after the other. This was because we preferred to use the same arm in order to avoid differences related to vascular stenosis or for other reasons related to different pulse transmission. However, it is unlikely that this methodology caused significant differences in blood pressure due to sudden changes in patients’ clinical conditions. Fourth, arterial stiffness is one of the conditions in which NIBP usually overestimates the true arterial pressure [6]. Many of our patients were affected by polydistrectual arteriopathy commonly characterized by increased arterial stiffness. This last could be considered a limitation of the study, although in those patients with underdamping/resonance, IBP resulted in the overestimation of NIBP. In light of this, it may be speculated that our results represent an underestimation, and that the actual IBP-NIBP differences could be even higher in patients with resonance. Finally, our observations cannot be generalized to include the patient population excluded from the study. Among these, we acknowledge obese patients for whom NIBP measurements were shown to be inaccurate [8], patients with arrhythmias, and patients with high doses of vasoconstrictors. Vasoconstrictors can be involved in underdamping/resonance phenomena. Nonetheless, our analysis did not identify norepinephrine administration as a correlated factor [20]. However, it should be underlined that the norepinephrine doses administered in both groups were quite low (≤0.02 μg/Kg/minute), and that the difference between groups was not statistically significant (*P* = 0.066). Finally, it is likely that low-dose norepinephrine may have played a minor role in the development of underdamping/resonance. It is reasonable to speculate that higher doses (for example, septic patients), may lead to different effects.

## Conclusions

In conclusion, IBP remains the gold standard for arterial pressure monitoring in critically ill patients. However, as faithful representation of arterial waveform and blood pressure values are the foremost prerequisites in intensive care, anesthesia, and modern medicine, the physician should be aware of the possibility that IBP can be overestimated in a consistent number of patients. NIBP may help to confirm/exclude the presence of underdamping phenomena and avoid inopportune treatments.

## Key messages

- Invasive blood pressure monitoring, free of artifacts, is the gold standard for arterial pressure measurement in critically ill patients
- Underdamping/resonance artifacts may alter blood pressure values in about one third of critically ill patients
- Physicians should always test arterial pressure waveform for underdamping/resonance artifacts
- The presence of a systolic invasive arterial pressure greater than 15 to 20 mmHg in respect of the non-invasive technique (or a dP/dt_MAX_ greater than 1.67 mmHg/msec) may help to identify the presence of underdamping phenomena

## Abbreviations

AR: amplitude ratio
CI: cardiac index
COPD: chronic obstructive pulmonary disease
Dia: diastolic
dP/dt_MAX_: maximal pressure-time ratio
Fn: natural frequency
IBP: invasive blood pressure
L: length
LoA: limits of agreement
NIBP: non-invasive blood pressure
NR-group: non resonance group
OR: odds ratio
r: radius
R-group: resonance group
ROC: receiving operating characteristic
Sys: systolic
β: damping coefficient
μ: viscosity
ρ: density
